# An Uncommon, Life-Threatening, Traumatic Hematoma in the Neck Area

**DOI:** 10.1155/2014/603742

**Published:** 2014-11-24

**Authors:** Michalis Peroulis, Georgios D. Lianos, Vasilios Nousias, Zoi Anastasiadi, Aikaterini Lianou, Christos Katsios, Miltiadis Matsagkas

**Affiliations:** Vascular Unit, Department of Surgery, University Hospital of Ioannina, 45110 Ioannina, Greece

## Abstract

It is well known that blunt neck trauma, when compared to a penetrating injury in the same anatomical area, is very rare. We report a case of an 81-year-old Caucasian woman with a blunt life-threatening neck trauma due to a bully goat. Although rare, direct evaluation should always be done in these cases because any misinterpretation may result in unfavorable outcomes. We have to highlight that close medical attention and prompt surgical treatment should be always considered in order to avoid dramatic consequences.

## 1. Introduction

It is reported that neck trauma accounts for 5–10% from all serious traumatic injuries and that blunt neck trauma is rare in comparison to a penetrating injury at the same anatomical region. The clinical symptomatology of a patient, following a blunt trauma in the neck area, includes dysphonia, hoarseness, dysphagia, odynophagia, dyspnea, pain, hemoptysis, and stridor [1].

The evaluation and management of a hemodynamically stable patient with penetrating neck injury has evolved considerably over the previous four decades. Algorithms developed in the 1970s focused on anatomic neck “zones” to distinguish triage pathways resulting from the operative constraints associated with very high or very low penetrations. During that era, mandatory endoscopy and angiography or mandatory neck exploration became popularized, the so-called “selective approach.” Currently, modern imaging techniques, including computed tomographic angiography (CTA), are widely available [2].

Blunt neck trauma has been a significant cause of morbidity and mortality for centuries. It is reported that apparently minor injuries can very quickly become life-threatening [3], since there is a plethora of vessels and delicate anatomical structures in this tight space. Many times, the rare and enigmatic nature of injury to this area may lead to the delay of the diagnosis. The trauma mechanisms include direct blows during sports, fast moving soccer balls, hockey pucks, and in extremely rare cases strangulation mechanisms with people or animals [4]. All experts in trauma units agree that although blunt neck trauma is in a minor way “amazing” in comparison with penetrating trauma, still these injuries may represent a fatal event [5].

## 2. Case Report

An 81-year-old Caucasian woman was presented to the emergency room of the University Hospital of Ioannina after a head-butt caused by a bully goat with a large hematoma at the right side of her neck and with severe dyspnea. From her medical history, she reports an “undefined” thyroid nodule discovered 15 years ago with gradually increasing dimensions over the last years. The patient was also hypertensive for many years.

**Figure 1:**
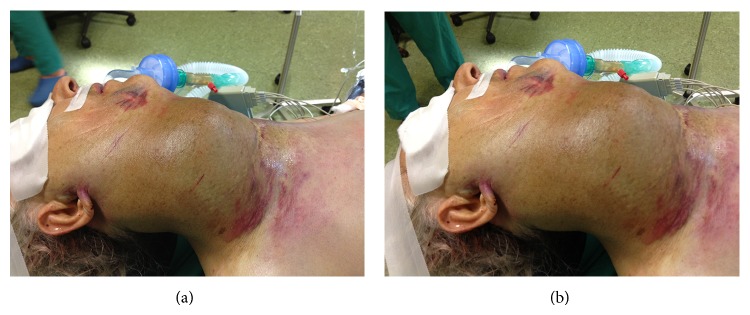
Patient intubated endotracheally.

Clinical examination revealed normal body temperature, heart rate at 118 beats per minute, and a blood pressure 95/75 mmHg. Physical examination of the neck revealed the presence of an extensive hematoma. The patient was barely speaking and her breath was noisy. Saturation of the arterial blood was 81%. The patient was intubated endotracheally (Figures 1(a) and 1(b)) in order to ensure a secure airway. She was then immediately transferred to the radiology department for an urgent CT scanning which revealed the extensive hematoma at the right area of the neck and a voluminous suspect thyroid nodule. There was no vertebral injury associated with this trauma. The performed angiography revealed a slight injury of the external carotid artery.

**Figure 2:**
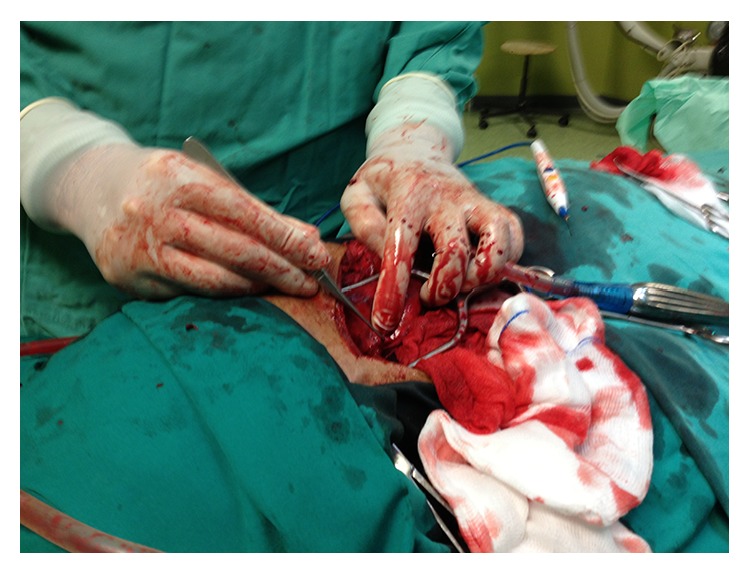
Ligated external carotid artery.

**Figure 3:**
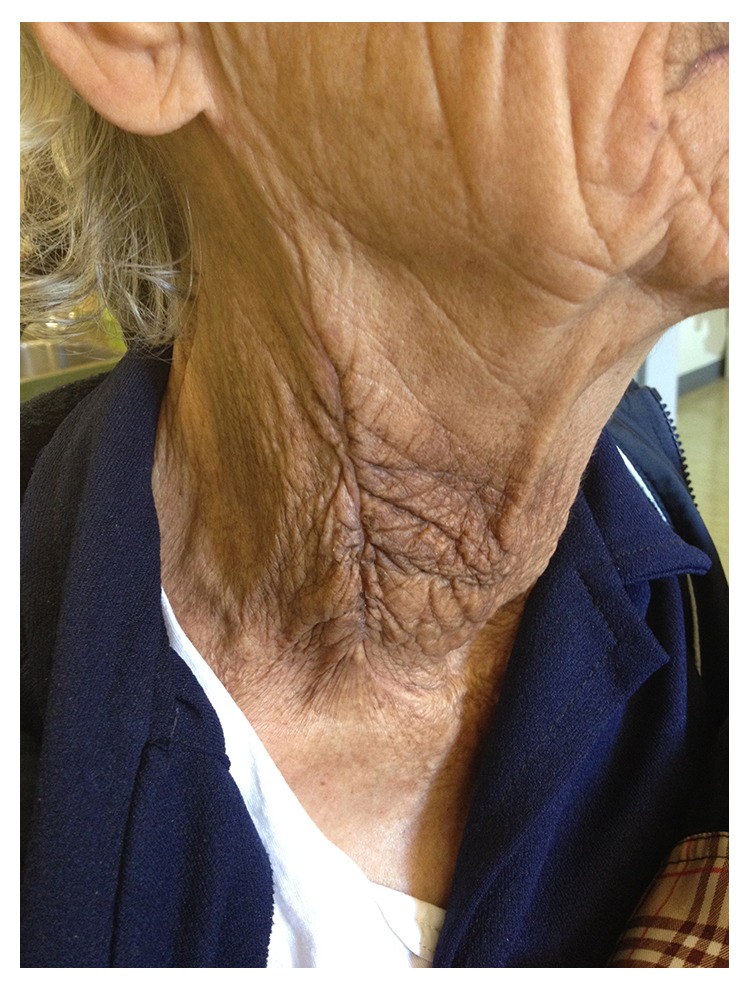
Our patient 6 months later.

The laboratory evaluation showed a hemoglobin level at 6,9 g/dL and hematocrit at 23%. The biochemistry profile of the patient was within normal limits. She was, then, transferred to the operating room where she was operated on by a vascular surgeon and a general surgeon. An extended incision along the anterior border of sternocleidomastoid muscle was made, which provided an excellent exposure of the damaged area. After the hematoma was drained the injured wall of the external carotid artery was revealed and the damaged arterial vessel was immediately ligated (Figure 2). Due to the presence of an oversized nodule on the thyroid gland, a partial thyroidectomy was performed. The postoperative period was uneventful and the patient was discharged from the hospital on the fourth postoperative day, in a good health. On a follow-up, six months later, the patient remained in an excellent condition (Figure 3).

## 3. Discussion

From the literature it is reported that neck trauma accounts for 5%–10% of all serious traumatic injuries, with a predominance observed in men between 20 and 30 years of age and with blunt neck trauma representing 3–11% of all head neck vascular injuries. Retrospective trials revealed that 30% of patients with no physical signs at the admission to the hospital had positive findings on surgical exploration. Schönholz et al. reported that physical examination is considered as an effective early and reliable marker to detect which patients will need a noninvasive or a later surgical investigation [6]. So, the attending physicians should always remain alert with these patients in order to avoid dramatic consequences.

Three mechanisms are reported to be responsible for dramatic consequences of the blunt neck trauma, external hemorrhage, extending soft tissue hematoma that distorts or causes real airway obstruction, and impaired cerebral circulation.

The critical and early steps in the management of these patients include the initial evaluation and stabilization of the trauma patient securing a free airway track, controlling the bleeding, immobilizing the cervical spine, and identifying promptly life-threatening situations from the thoracic cavity and/or the abdomen [7].

Blunt injuries to cervical arteries and veins, although extremely rare, are quite demanding from the attending physicians, since they are difficult to diagnose and treat in a quick and safe way. As for our paper, we have to highlight that we have performed a careful literature search and, as far as we know, this is the first successfully treated case of carotid artery injury due to animal thrust with life-threatening neck hematoma.

## 4. Conclusion

A blunt trauma in the neck area requires, most of the times, high index of suspicion on the part of the medical staff of the emergency room. Their immediate priority is to secure a patent airway track and keep the patient hemodynamically stable. Prompt surgical exploration of the carotid artery should be considered in case that the patient is deteriorating hemodynamically.
